# Downregulation of BRAF activated non-coding RNA is associated with poor prognosis for non-small cell lung cancer and promotes metastasis by affecting epithelial-mesenchymal transition

**DOI:** 10.1186/1476-4598-13-68

**Published:** 2014-03-21

**Authors:** Ming Sun, Xiang-Hua Liu, Ke-Ming Wang, Feng-qi Nie, Rong Kong, Jin-song Yang, Rui Xia, Tong-Peng Xu, Fei-Yan Jin, Zhi-Jun Liu, Jin-fei Chen, Er-Bao Zhang, Wei De, Zhao-Xia Wang

**Affiliations:** 1 Department of Biochemistry and Molecular Biology, Nanjing Medical University, Nanjing 210029, People’s Republic of China; 2 Department of Oncology, Second Affiliated Hospital, Nanjing Medical University, Nanjing, Jiangsu 210011, People’s Republic of China; 3 Department of Oncology, First Affiliated Hospital, Nanjing Medical University, Nanjing, People’s Republic of China; 4 Department of Oncology, Nanjing First Hospital, Nanjing Medical University, Nanjing, P. R. China

## Abstract

**Background:**

Recent evidence indicates that long noncoding RNAs (lncRNAs) play a critical role in the regulation of cellular processes, such as differentiation, proliferation and metastasis. These lncRNAs are found to be dysregulated in a variety of cancers. BRAF activated non-coding RNA (BANCR) is a 693-bp transcript on chromosome 9 with a potential functional role in melanoma cell migration. The clinical significance of BANCR, and its’ molecular mechanisms controlling cancer cell migration and metastasis are unclear.

**Methods:**

Expression of BANCR was analyzed in 113 non-small cell lung cancer (NSCLC) tissues and seven NSCLC cell lines using quantitative polymerase chain reaction (qPCR) assays. Gain and loss of function approaches were used to investigate the biological role of BANCR in NSCLC cells. The effects of BANCR on cell viability were evaluated by MTT and colony formation assays. Apoptosis was evaluated by Hoechst staining and flow cytometry. Nude mice were used to examine the effects of BANCR on tumor cell metastasis *in vivo*. Protein levels of BANCR targets were determined by western blotting and fluorescent immunohistochemistry.

**Results:**

BANCR expression was significantly decreased in 113 NSCLC tumor tissues compared with normal tissues. Additionally, reduced BANCR expression was associated with larger tumor size, advanced pathological stage, metastasis distance, and shorter overall survival of NSCLC patients. Reduced BANCR expression was found to be an independent prognostic factor for NSCLC. Histone deacetylation was involved in the downregulation of BANCR in NSCLC cells. Ectopic expression of BANCR impaired cell viability and invasion, leading to the inhibition of metastasis *in vitro* and *in vivo*. However, knockdown of BANCR expression promoted cell migration and invasion *in vitro*. Overexpression of BANCR was found to play a key role in epithelial-mesenchymal transition (EMT) through the regulation of E-cadherin, N-cadherin and Vimentin expression.

**Conclusion:**

We determined that BANCR actively functions as a regulator of EMT during NSCLC metastasis, suggesting that BANCR could be a biomarker for poor prognosis of NSCLC.

## Background

Non-small cell lung cancers (NSCLCs), including adenocarcinomas and squamous cell carcinomas, are the predominant forms of lung cancer and account for the majority of cancer deaths worldwide [1]. Despite recent advances in clinical and experimental oncology, the prognosis of lung cancer remains poor, with a 5-year overall survival rate of around 11% [2]. A continuing problem of NSCLC tumorigenesis is the metastasis of cancer cells, which is the main cause of death in patients. Thus, a detailed understanding of the mechanisms and molecular pathways activated in metastatic cells is crucial in identifying new treatment options for anticancer therapy that target metastasis.

The invasion and metastasis of cancer cells are landmark events that involve many changes in cellular behavior, and lead to different steps of the metastatic cascade [3,4]. One of the most crucial steps in the metastatic cascade is the acquisition of invasive capabilities, including turnover of cell-cell junctions, degradation of the cell matrix, and activation of pathways that control cytoskeletal dynamics in cancer cells. This process is accompanied by multiple changes in gene expression, such as the loss of epithelial markers and a gain in mesenchymal markers [5,6]. Over the past decade, cell and tumor biologists have identified the key role of epithelial-mesenchymal transition (EMT) in cancer cell metastasis, a biological process where epithelial cells lose their polarity and undergo transition into a mesenchymal phenotype [7].

EMT enhances tumor cell invasion in response to environmental triggers, and augments invasive functions by promoting Rac-dependent mesenchymal migration, and also contributes to cell growth and survival [8,9]. Important hallmarks of EMT include the loss of E-cadherin expression, and increased expression of non-epithelial cadherins, such as N-cadherin. The loss of E-cadherin expression is a fundamental event in EMT, and a crucial step in the progression of papillomas to invasive carcinomas [10]. To date, substantial effort has been devoted to understanding how EMT is regulated during cancer progression. It has been verified that EMT can be initiated by external signals, such as hepatocyte growth factor (HGF), epidermal growth factor (EGF), transforming growth factor (TGF)-b, and fibroblast growth factor (FGF) [11]. In addition to these signaling pathways triggered by membrane receptors, recent studies have highlighted the importance of noncoding RNAs in the regulation of the epithelial phenotype by controlling EMT inducers. The miR-200 family has been found to control EMT by downregulating the expression of Zeb factors [12]. Furthermore, the long noncoding RNA (lncRNA) MALAT-1 promoted EMT by regulating ZEB1, ZEB2 and Slug expression, and activating Wnt signaling [13].

The lncRNAs are important new members of the ncRNA family, that are greater than 200 nt, and are unable to be translated into proteins. These lncRNAs are often expressed in a spatial- and temporal-specific pattern. Although very few lncRNAs have been characterized in detail, they have been found to participate in a large range of biological processes, including modulation of apoptosis and invasion, reprogramming stem cell pluripotency, and parental imprinting. These findings indicate that lncRNAs play a major role in the regulation of the eukaryotic genome [14-16]. Researchers have linked the dysregulation of lncRNAs with diverse human diseases, in particular cancers [17-19]. Therefore, identification of cancer-associated lncRNAs and investigation of their molecular and biological functions in controlling EMT are important in understanding the molecular biology of NSCLC metastasis and progression.

BRAF-activated non-coding RNA (BANCR), an 693-bp lncRNA on chromosome 9 was firstly found by Ross J. Flockhart et.al via RNA-seq screen for transcripts affected by the expression of the oncogene *BRAF*^*V600E*^. BANCR is overexpressed in melanoma cells and crucial for melanoma cell migration [20]. In this study, we investigated the effects of BANCR expression on NSCLC cell phenotypes *in vitro* and *in vivo*. Moreover, we also showed that alteration of BANCR expression can influence E-cadherin, N-cadherin and Vimentin protein levels, which indicated that BANCR affected NSCLC cells invasion and metastasis partly via epithelial-mesenchymal transition. This study advances our understanding of the role of lncRNAs, such as BANCR as a regulator of pathogenesis of NSCLC and facilitate the development of lncRNA-directed diagnostics and therapeutics.

## Results

### BANCR expression was downregulated and correlated with poor prognosis of NSCLC

**Figure 1:**
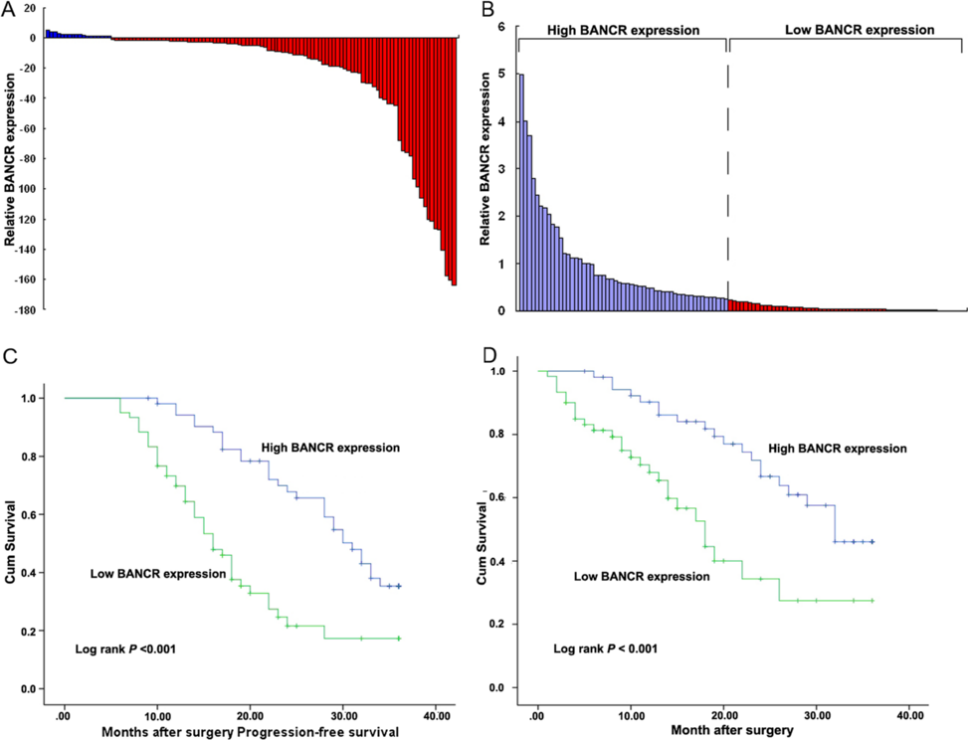
**Relative BANCR expression in NSCLC tissues and its clinical significance. (A)** Relative expression of BANCR in NSCLC tissues (*n* = 113) compared with corresponding non-tumor tissues (*n* = 113). BANCR expression was examined by qPCR and normalized to GAPDH expression. Results were presented as the fold-change in tumor tissues relative to normal tissues. **(B)** BANCR expression was classified into two groups. **(C, D)** Kaplan–Meier disease-free survival and overall survival curves according to BANCR expression levels. *P < 0.05, **P < 0.01.

BANCR expression levels were investigated in 113 paired NSCLC samples and adjacent histologically normal tissues using quantitative polymerase chain reaction (qPCR) assays. BANCR expression was significantly downregulated (*P* < 0.01) in 79% (89/113) of cancerous tissues compared with normal tissues (Figure 1A). BANCR expression levels in NSCLC were significantly correlated with tumor size (*p* = 0.001), advanced pathological stage (*p* < 0.001), and lymph node metastasis (*p* = 0.001). However, BANCR expression was not associated with other parameters such as gender (*p* = 0.232) and age (*p* = 0.616) in NSCLC (Table 1). The clinical data for all patients were summarized in Additional file 1: Table S1.

**Table 1:** Correlation between BANCR expression and clinicopathological characteristics of NSCLC patients (n = 113)

**Characteristics**	**BANCR**		**P**
	**High no. cases (%)**	**Low no. cases (%)**	**Chi-squared test P-value**
**Age(years)**			0.616
≤65	29(54.7)	30(50.0)	
>65	24(45.3)	30(50.0)	
**Gender**			0.232
Male	35(66.0)	33(55.0)	
Female	18(34.0)	27(45.0)	
**Histological subtype**			0.466
Squamous cell carcinoma	30(56.6)	38(63.3)	
Adenocarcinoma	23(43.4)	22(36.7)	
**TNM Stage**			<0.001*
Ia + Ib	25(47.2)	9(15.0)	
IIa + IIb	17(32.1)	21(35.0)	
IIIa	11(20.7)	30(50.0)	
**Tumor size**			0.001*
≤5cm	35(66.0)	21(35.0)	
>5cm	18(34.0)	39(65.0)	
**Lymph node metastasis**			0.001*
Negative	34(64.2)	20(33.3)	
Positive	19(35.8)	40(66.7)	
**Smoking History**			0.127
Smokers	39(64.2)	36(60.0)	
Never Smokers	14(35.8)	24(40.0)	

* Overall P < 0.05.

### Association of BANCR expression with patients’ survival

Kaplan-Meier survival analysis was conducted to investigate the correlation between BANCR expression and NSCLC patient prognosis. According to relative BANCR expression in tumor tissues, the 113 NSCLC patients were classified into two groups: the high BANCR group (*n* = 53, fold-change ≤ 4); and the low BANCR group (*n* = 60, fold-change ≥4) (Figure 1B). With respect to progression-free survival (PFS), this was 35.3% for the high BANCR group, and 17.2% for the low BANCR group. Median survival time for the high BANCR group was 31 months, and 16 months for the low BANCR group (Figure 1C). The overall survival rate over 3 years for the high BANCR group was 46%, and 27.5% for the low BANCR group. Median survival time for the high BANCR group was 32 months, and 18 months for the low BANCR group (Figure 1D).

Univariate analysis identified three prognostic factors: lymph node metastasis; TNM stage; and BANCR expression level. Other clinicopathological features such as gender and age were not statistically significant prognosis factors (Table 2). Multivariate analysis of the three prognosis factors confirmed that a low BANCR expression level was an independent predictor of poor survival for NSCLC (*p* = 0.031), in addition to TNM stage (*p* = 0.038) (Table 2).

**Table 2:** Univariate and multivariate analysis of overall survival in NSCLC patients (n = 113)

**Variables**	**Univariate analysis**			**Multivariate analysis**		
	**HR**	**95% CI**	**p value**	**HR**	**95% CI**	**p value**
**Age**	1.257	0.712-2.219	0.431			
**Gender**	1.185	0.670-2.098	0.559			
**Smoker**	1.120	0.842-1.491	0.436			
**Histological subtype**	0.982	0.738-1.307	0.902			
**Chemotherapy**	0.787	0.587-1.055	0.110			
**Tumor size**	1.233	0.926-1.640	0.151			
**Lymph node metastasis**	0.424	0.235-0.764	0.004*	0.577	0.311-1.071	0.081
**TNM stage (I vs. II or IIIa)**	0.320	0.149-0.685	0. 003*	0.431	0.195-0.954	0.038*
**BANCR expression**	0.367	0.201-0.669	0. 001*	0.496	0.262-0.938	0.031*

HR, hazard ratio; 95 % CI, 95 % confidence interval, * Overall P < 0.05.

### Histone deacetylation is involved in the downregulation of BANCR

**Figure 2:**
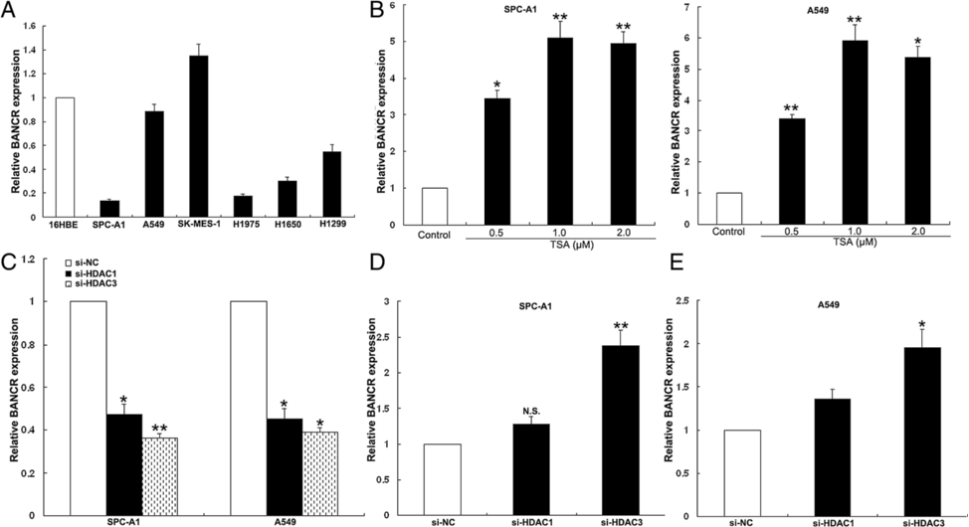
**Histone deacetylation is involved in BANCR downregulation. (A)** BANCR expression levels of NSCLC cell lines (A549, SPC-A1, H1299, H1650, H1975 and SK-MES-1) compared with that in normal human bronchial epithelial cells (16HBE). **(B)** qPCR analysis of BANCR expression levels following the treatment of SPC-A1 and A549 cells with TSA. **(C)** qPCR analysis of HDAC2 and HDAC3 expression levels following the treatment of SPC-A1 and A549 cells with si-HDAC2 or si-HDAC3**.(D, E)** qPCR analysis of BANCR expression levels following the treatment of SPC-A1 and A549 cells with si-HDAC1 and si-HDAC3.

Expression levels of BANCR in NSCLC cell lines were determined by qPCR. Compared with that in 16HBE cells, relative expression levels of BANCR were reduced in NSCLC cells (Figure 2A). Because of the different expression patterns for BANCR in NSCLC and melanomas, we investigated the mechanisms controlling tissue-specific expression of BANCR. We analyzed the promoter region of BANCR, and found there were no CpG islands (data not shown). Histone protein modification was thought to play an important role in the transcription of lncRNAs; however, knockdown of two core subunits of polycomb repressive complex 2 (SUZ12 and EZH2) had no influence on BANCR expression (Additional file 2: Figure S1A).

We observed that BANCR expression was upregulated in SPC-A1 and A549 cells, following treatment with the histone deacetylase (HDAC) inhibitor trichostatin A (TSA) (Figure 2B). We sought to determine whether repression of BANCR was mediated by HDACs. Specific anti-HDAC1 and HDAC3 siRNAs were transfected into NSCLC cells, and HDAC1 and HDAC3 expression was significantly decreased (Figure 2C). Expression levels of BANCR were significantly upregulated in cells transfected with si-HDAC3. Transfection with the scrambled siRNA or si-HDAC1 did not induce BANCR expression (Figure 2D and E). Moreover, the HDAC3 expression was upregulated in NSCLC cells and negatively correlated with BANCR expression (Additional file 2: Figure S1B). Furthermore, NSCLC cells were treated with RGFP966, which is an seletive inhibitor for HDAC3 with an IC50 of 0.08μM and no effective inhibition of other HDACs at concentrations up to 15μM. The results of qPCR showed that the expression of BANCR was upregulated in NSCLC cells after treated with RGFP966 when compared with control cells (Additional file 2: Figure S1C). These data indicate that HDAC3 knockdown induced BANCR increase may be due to the inhibition of HDAC3 enzymatic activity.

### BANCR inhibits NSCLC cell viability and induces apoptosis

**Figure 3:**
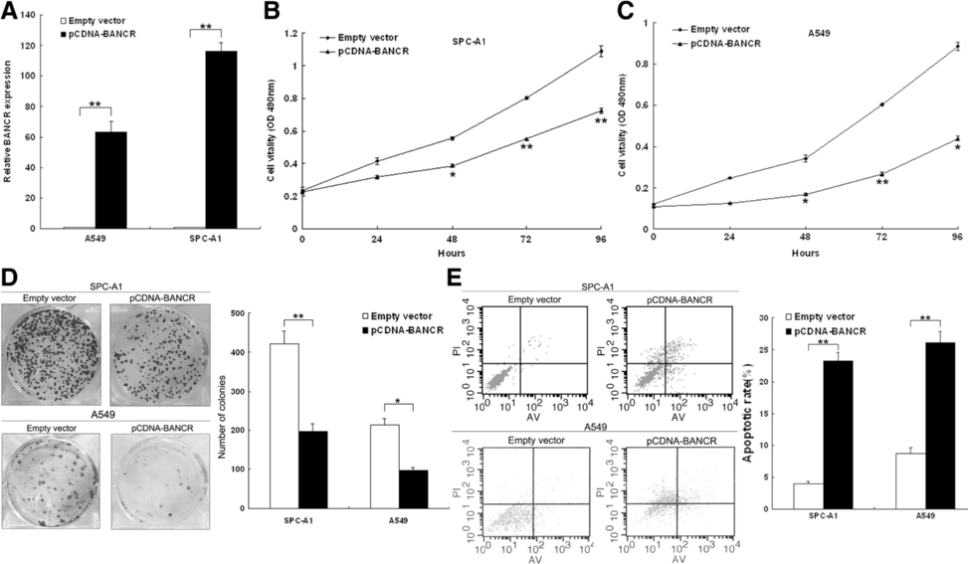
**Effects of BANCR on NSCLC cell viability and apoptosis *in vitro*. (A)** SPC-A1 and A549 cells were transfected with pCDNA-BANCR. **(B, C)** MTT assays were used to determine the cell viability for pCDNA-BANCR-transfected SPC-A1 and A549 cells. Values represented the mean ± s.d. from three independent experiments. **(D)** Colony-forming assays were conducted to determine the proliferation of pCDNA-BANCR-transfected SPC-A1 and A549 cells. **(E)** Apoptosis was determined by flow cytometry. UL, necrotic cells; UR, terminal apoptotic cells; LR, early apoptotic cells. *P < 0.05 and **P < 0.01.

To assess the biological role of BANCR in NSCLC, we investigated the effects of BANCR over-expression on the viability and apoptosis of SPCA1 or A549 cells. Our qPCR results revealed that BANCR expression was significantly upregulated compared with that in control cells (Figure 3A). MTT assay results showed that the growth of SPC-A1 and A549 cells transfected with pCDNA-BANCR was impaired compared with that for control cells (Figure 3B and C). Colony formation assay results revealed that clonogenic survival was inhibited following overexpression of BANCR in SPC-A1 and A549 cells (Figure 3D). Flow cytometry analysis of SPC-A1 and A549 cells showed that upregulation of BANCR expression promoted apoptosis in comparison with that in control cells (Figure 3E).

### BANCR inhibits migration and invasion of NSCLC cells

**Figure 4:**
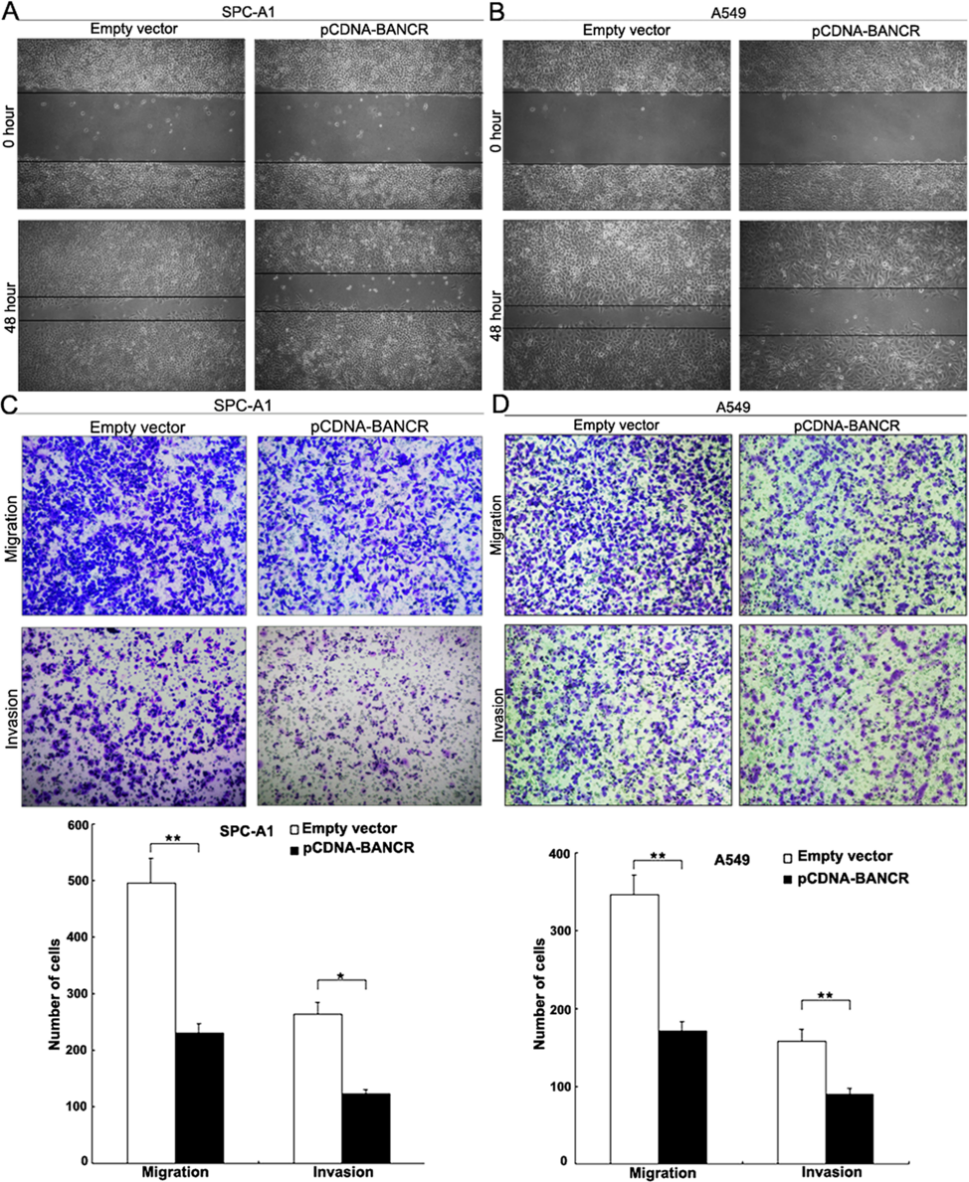
**Effects of BANCR on NSCLC migration and invasion *in vitro*.** SPC-A1 and A549 cells were transfected with pCDNA-BANCR. **(A, B)** Wound-healing assays were used to investigate the migratory ability of NSCLC cells. **(C, D)** Transwell assays were used to investigate the changes in migratory and invasive abilities of NSCLC cells. *P < 0.05 and **P < 0.01.

The wound healing assay results showed that cells transfected with pCDNA-BANCR resulted in a slower closing of scratch wounds compared with that for control cells (Figure 4A and B). We evaluated cancer cell invasion through matrigel, and migration through transwells. Increased BANCR expression levels impeded the migration of SPC-A1 and A549 cells by approximately 64% compared with controls (Figure 4C and D). Similarly, invasion of SPC-A1 and A549 cells was also reduced by 59% following upregulation of BANCR expression.

### Knockdown of BANCR expression promotes NSCLC cells invasion

**Figure 5:**
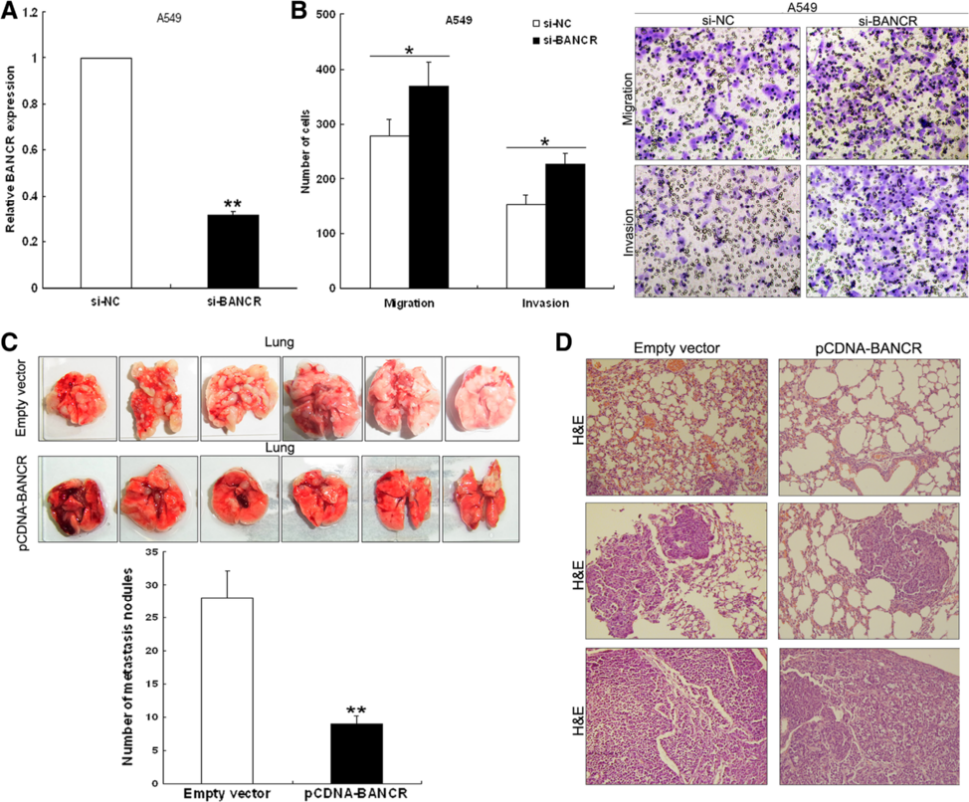
**Effects of BANCR overexpression on tumor metastasis *in vivo*. (A)** BANCR expression levels were determined by qPCR following the treatment of A549 cells with si-BANCR. **(B)** Transwell assays were conducted to determine the migratory and invasive abilities of si-BANCR-transfected A549 cells. Analysis of an experimental metastasis animal model was performed by injecting *BANCR*-overexpressing SPC-A1 cells into nude mice. **(C)** Lungs from mice in each experimental group, with the numbers of tumor nodules on lung surfaces were shown. **(D)** Visualization of the entire lung, and HE-stained lung sections. **P < 0.01.

To determine whether inhibition of BANCR expression could promote NSCLC cells viability and invasion, we performed targeted knockdown of BANCR expression using RNA interference (RNAi) in A549 cells (Figure 5A). MTT assays revealed that downregulation of BANCR expression did not affect cell viability (data not shown). However, decreased BANCR expression levels promoted A549 cell migration and invasion in *vitro* (Figure 5B).

### BANCR suppresses NSCLC cell metastasis *in vivo*

To validate the effects of BANCR on the metastasis of NSCLC cells *in vivo*, SPCA1 cells stably transfected with pCDNA-BANCR were injected into nude mice. Metastatic nodules on the surface of lungs were counted after 7 weeks. Ectopic overexpression of BANCR resulted in a reduction of the number of metastatic nodules compared with those in the control group (Figure 5C). This difference was further confirmed following examination of the entire lungs, and through hematoxylin and eosin (HE) staining of lung sections (Figure 5D). Our *in vivo* data complemented the results of functional *in vitro* studies involving BANCR.

### BANCR influences NSCLC cell EMT

**Figure 6:**
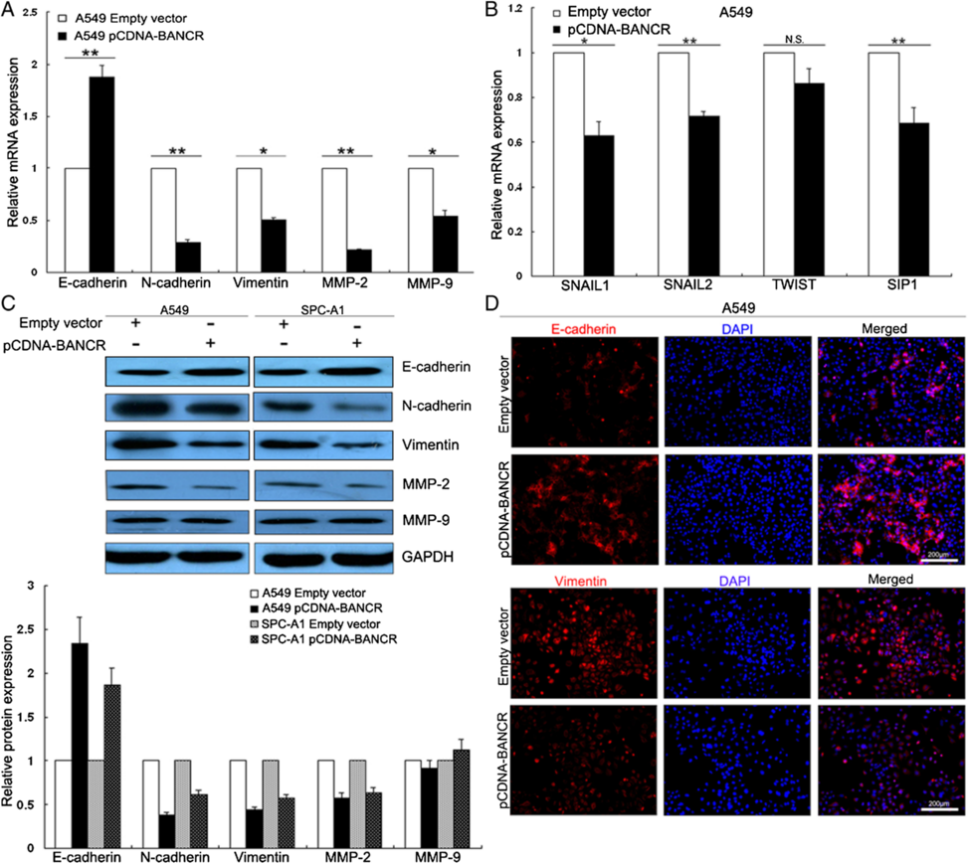
**BANCR overexpression suppresses NSCLC cell invasion and metastasis by affecting EMT. (A, B)** Analysis of E-cadherin, N-cadherin, Vimentin, MMP-2, MMP-9, SNAIL1, SNAIL2, TWIST and SIP1 expression in A549 cells treated with pCDNA-BANCR. **(C,D)** Analysis of E-cadherin and Vimentin expression in A549 cells treated with pCDNA-BANCR by western blot and immunofluorescence. All experiments were performed in triplicate with three technical replicates. *P < 0.05 , **P < 0.01.

We conducted qPCR and western blotting assays to detect the expression of EMT-induced markers (E-cadherin, N-cadherin and Vimentin) in cells over-expressing BANCR. Our findings showed that increased BANCR expression levels induced E-cadherin expression, while decreased N-cadherin, Vimentin and MMP-2 expression (Figure 6A). Simultaneously, upregulation of BANCR expression led to decreased SNAIL1, SNAIL2, and SIP1 expression (Figure 6B). Western blotting and immunofluorescence analysis also revealed that enhanced BANCR expression stimulated E-cadherin expression and reduced Vimentin expression in NSCLC cells (Figure 6B and C).

## Discussion

Recent evidence has shown that ncRNAs play an important role in cancer pathogenesis, and could provide new insights into the biology of this disease [21,22]. Over the past decade, microRNAs (miRNAs) have moved to the forefront of ncRNA research in NSCLC. However, lncRNAs in NSCLC are still an emerging field, with only a handful of lncRNAs involved in NSCLC tumorigenesis. One of these lncRNAs is metastasis-associated lung adenocarcinoma transcript 1 (MALAT1). MALAT1, also known as NEAT2 (nuclear-enriched abundant transcript 2), is a highly conserved nuclear lncRNA and a predictive marker for metastasis development in lung cancer [23].

In this study, we found that the expression of another lncRNA, BANCR, was significantly downregulated in NSCLC tissues. Specifically, BANCR expression was significantly lower at the later stages of tumor development, and in tumors that had undergone extensive metastasis. Moreover, the overall survival time of patients with lower BANCR expression levels was significantly shorter than that for patients with higher BANCR expression levels. Our results indicate that BANCR expression provided a significant, independent predictive value for TNM stage (*P* = 0.038). We demonstrated that upregulation of BANCR expression led to the significant inhibition of cell viability, migration, invasion, and promotion of apoptosis. Knockdown of BANCR expression promoted cell migration and invasion. BANCR induced cell apoptosis may be partly via P53, which could contribute to the less cells in migration and invasion; however, the impaired migration and invasion ability is the main reason which could be supported by wound-healing assay. Moreover, increased BANCR expression levels resulted in a significant reduction in the number of metastatic nodules on the lungs in *vivo*. These findings suggest that BANCR plays a direct role in the modulation of cell metastasis and NSCLC progression, and may be useful as a novel prognostic or progression marker for NSCLC.

Tumor development and progression is precisely regulated by several subsets of genes that act by either silencing tumor suppressor genes or activating oncogenes [24]. Tumor suppressor genes can negatively regulate cell proliferation by inducing growth arrest and inhibiting cell invasion. In cancer cells, tumor suppressor genes are usually silenced by genetic or epigenetic alterations [25]. Whether epigenetic regulatory factors, such as histone acetylation or DNA methylation, manipulate the expression of lncRNAs remains unclear. Hypermethylation of the promoter or the intergenic differentially methylated region has been found to contribute to reduced lncRNA MEG3 expression in tumors, indicating that epigenetic regulation is also involved in the expression of these genes [26,27]. Our findings highlight that histone acetylation is a key factor in controlling lncRNA BANCR expression. These results, along with those from a recent study [28], highlight the role of epigenetics in regulating lncRNA transcription.

To explore the molecular mechanism through which BANCR contributes to the invasion and metastasis of NSCLC, we investigated potential target proteins involved in cell motility and matrix invasion. Hallmarks of EMT are the loss of E-cadherin expression, and aberrant expression of N-cadherin and Vimentin [29-32]. Therefore, we determined the protein levels of these EMT-induced markers following BANCR overexpression. Our results indicated that inhibitory effects on cell migration and invasion were associated with EMT. Matrix metalloproteases (MMPs) are also important to many aspects of biology, ranging from cell proliferation, differentiation and remodeling of the extracellular matrix (ECM), to vascularization and cell migration. Upregulation of BANCR expression in NSCLC cells led to a significant decrease in MMP2 protein levels. Our findings demonstrated that BANCR mediated NSCLC cell migration, invasion and metastasis suppression, which possibly also affected EMT.

As a central differentiation process, EMT allows for remodeling of tissues during the early stages of embryogenesis, and is implicated in the promotion of tumor cell invasion and metastasis [7,33]. It has been proposed, and supported by many studies, that EMT could be a potent mechanism for promoting the detachment of cancer cells from primary tumors. A characteristic of cells that undergo EMT is increased expression levels of N-cadherin and Vimentin, and a loss of E-cadherin expression. Importantly, EMT has been reported to be associated with poor clinical outcome in NSCLC [34,35]. Therefore, lncRNAs as regulators of EMT might be suitable candidates for intervention in the treatment of cancer.

Although only a small number of functional lncRNAs have been well characterized to date, they have been shown to regulate gene expression at various levels, including chromatin modification, transcription and post-transcriptional processing. Hox transcript antisense intergenic RNA (HOTAIR) is one of the most studied lncRNAs involved in chromatin modification, which can target PRC2 genome-wide to alter H3K27 methylation and gene expression patterns [22]. A muscle-specific lncRNA, linc-MD1 may function as competing endogenous RNAs (ceRNAs) to sponge miRNAs, thereby modulating the derepression of miRNA targets and impose an additional level of post-transcriptional regulation [36]. Here, although we observed BANCR overexpression induced NSCLC cells apoptosis and regulate EMT phenotype, the possible mechanisms that underlie such regulatory behaviors still remain to be fully understood. Further investigation of BANCR molecular and biological functions in controlling EMT will undoubtedly be important in understanding the molecular biology of NSCLC metastasis and progression.

## Conclusions

The expression of BANCR was significantly decreased in NSCLC tissues, suggesting that its downregulation may be a negative prognostic factor for NSCLC patients, and indicative of poor survival rates and a higher risk for cancer metastasis. We showed that BANCR possibly regulates the invasive and metastatic ability of NSCLC cells, partially through regulation of EMT. Our findings further the understanding of NSCLC pathogenesis and development, and facilitate the development of lncRNA-directed diagnostics and therapeutics against cancers. However, the molecular mechanisms through which BACNR regulates EMT requires further investigation.

## Methods

### Tissue collection

We obtained 113 paired NSCLC and adjacent non-tumor lung tissues from patients who underwent surgery at Jiangsu Province Hospital between 2008 and 2010, and were diagnosed with NSCLC (stages I, II, and III) based on histopathological evaluation. Clinicopathological characteristics, including tumor-node-metastasis (TNM) staging, were recorded. No local or systemic treatment was conducted in these patients before surgery. All collected tissue samples were immediately snap-frozen in liquid nitrogen and stored at –80°C until required. Our study was approved by the Research Ethics Committee of Nanjing Medical University, China. Written informed consent was obtained from all patients.

### Cell lines

Five NSCLC adenocarcinoma cell lines (A549, SPC-A1, NCI-H1975, NCI-H1299, and NCI-H1650), a NSCLC squamous carcinomas cell line (SK-MES-1), and a normal human bronchial epithelial cell line (16HBE) were purchased from the Institute of Biochemistry and Cell Biology of the Chinese Academy of Sciences (Shanghai, China). A549, SK-MES-1, NCI-H1975, NCI-H1299, NCI-H1650 and 16HBE cells were cultured in RPMI 1640; SPC-A1 cells were cultured in DMEM (GIBCO-BRL) medium supplemented with 10% fetal bovine serum (FBS), 100 U/ml penicillin and 100 mg/ml streptomycin (Invitrogen, Carlsbad, CA, USA) at 37ºC/5% CO_2_.

### RNA extraction and qPCR assays

Total RNA was isolated with TRIzol reagent (Invitrogen) according to the manufacturer’s instructions. Total RNA (500 ng) was reverse transcribed in a final volume of 10 μl using random primers under standard conditions for the PrimeScript RT reagent Kit (TaKaRa, Dalian, China). We used the SYBR Premix Ex Taq (TaKaRa, Dalian, China) to determine BANCR expression levels, following the manufacturer’s instructions. Results were normalized to the expression of glyceraldehyde-3-phosphate dehydrogenase (*GAPDH*). The specific primers used are presented in Additional file 3: Table S2. The qPCR assays were conducted on an ABI 7500, and data collected with this instrument. Our qPCR results were analyzed and expressed relative to threshold cycle (CT) values, and then converted to fold changes.

### Plasmid generation

The BANCR sequence was synthesized and subcloned into the pCDNA3.1 (Invitrogen, Shanghai, China) vector. Ectopic expression of BANCR was achieved through pCDNA-BANCR transfection, with an empty pCDNA3.1 vector used as a control. The expression levels of BANCR were detected by qPCR.

### Cell transfection

Plasmid vectors (pCDNA3.1-BANCR and pCDNA3.1) for transfection were prepared using DNA Midiprep or Midiprep kits (Qiagen, Hilden, Germany), and transfected into SPC-A1 or A549 cells. The siRNAs si-HDAC1, si-HDAC3, si-BANCR or si-NC were transfected into SPC-A1 or A549 cells (Additional file 3: Table S2). A549 and SPC-A1 cells were grown on six-well plates to confluency and transfected using Lipofectamine 2000 (Invitrogen) according to the manufacturer’s instructions. At 48 h post-transfection, cells were harvested for qPCR or western blot analysis.

### Cell viability assays

Cell viability was monitored using a Cell Proliferation Reagent Kit I (MTT) (Roche Applied Science). The A549 cells transfected with si-BANCR (3000 cells/well), and A549 or SPC-A1 cells transfected with pCDNA-BANCR were grown in 96-well plates. Cell viability was assessed every 24 h following the manufacturer’s protocol. All experiments were performed in quadruplicate. For colony formation assays, pCDNA-BANCR-transfected SPC-A1 or A549 cells (n = 500) were placed in a 6-well plates and maintained in media containing 10% FBS. The medium was replaced every 4 days; after 14 days, cells were fixed with methanol and stained with 0.1% crystal violet (Sigma-Aldrich). Visible colonies were then counted. For each treatment group wells were assessed in triplicate.

### Flow cytometry analysis of apoptosis

SPC-A1 and A549 cells were harvested at 48 h post-transfection by trypsinization. After staining with FITC-Annexin V and propidium iodide, cells were analyzed by flow cytometry (FACScan; BD Biosciences) using CellQuest software (BD Biosciences). Cells were discriminated into viable cells, dead cells, early apoptotic cells, and apoptotic cells. The ratio of early apoptotic cells was compared to that for controls from each experiment. All samples were assayed in triplicate.

### Wound-healing assay

For the wound-healing assay, 3 × 10^5^ cells were seeded in 6-well plates, cultured overnight, and transfected with pCDNA-BANCR or the control vector. Once cultures reached 85% confluency, the cell layer was scratched with a sterile plastic tip and washed with culture medium, then cultured for 48 h with medium containing 1% FBS. At different time points, images of the plates were acquired using a microscope. The distance between the two edges of the scratch was measured using Digimizer software system.

### Cell migration and invasion assays

For the migration assays, at 48 h post-transfection, 5 × 10^4^ cells in serum-free media were placed into the upper chamber of an insert (8-μm pore size; Millipore). For the invasion assays, 1 × 10^5^ cells in serum-free medium were placed into the upper chamber of an insert coated with Matrigel (Sigma-Aldrich). Medium containing 10% FBS was added to the lower chamber. After incubation for 24 h, the cells remaining on the upper membrane were removed with cotton wool. Cells that had migrated or invaded through the membrane were stained with methanol and 0.1% crystal violet, imaged, and counted using an IX71 inverted microscope (Olympus, Tokyo, Japan). Experiments were independently repeated three times.

### Tail vein injections into athymic mice

Athymic male mice (4-weeks-old) were purchased from the Animal Center of the Chinese Academy of Science (Shanghai, China) and maintained in laminar flow cabinets under specific pathogen-free conditions. SPC-A1 cells transfected with pCDNA-BANCR or the empty vector were harvested from 6-well plates, washed with phosphate-buffered saline (PBS), and resuspended at 2 × 10^7^ cells/ml. Suspended cells (0.1 ml) were injected into the tail veins of 9 mice, which were sacrificed 7 weeks after injection. The lungs were removed and photographed, and visible tumors on the lung surface were counted. This study was carried out in strict accordance with the Guide for the Care and Use of Laboratory Animals of the National Institutes of Health. Our protocol was approved by the Committee on the Ethics of Animal Experiments of Nanjing Medical University (Permit Number: 200933). All surgery was performed under sodium pentobarbital anesthesia, and all efforts were made to minimize suffering [37].

### Western blotting analysis

Cells were lysed using RIPA protein extraction reagent (Beyotime, Beijing, China) supplemented with a protease inhibitor cocktail (Roche, CA, USA) and phenylmethylsulfonyl fluoride (Roche). The concentration of proteins was determined using the Bio-Rad protein assay kit. Protein extracts (50 μg) were separated by 10% sodium dodecyl sulfate-polyacrylamide gel electrophoresis (SDS-PAGE), then transferred to nitrocellulose membranes (Sigma) and incubated with specific antibodies. ECL chromogenic substrate was used to visualize the bands and the intensity of the bands was quantified by densitometry (Quantity One software; Bio-Rad), with GAPDH used as a control. Antibodies (1:1000 dilution) against E-cadherin and N-cadherin were purchased from BD. Antibodies against vimentin, MMP-2, and MMP-9 were purchased from Cell Signaling Technology (MA, USA).

### Fluorescence immunohistochemistry

Cells were fixed in 4% paraformaldehyde following a standard protocol. Mouse anti-E-cadherin and -N-cadhherin polyclonal antibodies (1:100; BD) were used as primary antibodies, with TRITC-labeled anti-Rabbit IgG (1:200; Sigma) used as a secondary antibody. Sections were mounted onto slides using Gel Mount Aqueous Mounting Medium (G0918, Sigma) and examined with an Olympus BX51 microscope (Olympus Optical, Tokyo, Japan).

### Statistical analysis

Student’s *t*-test (2-tailed), one-way ANOVA, and the Mann–Whitney U test were used to analyze data, along with SPSS 16.0 (IBM, IL, USA). *P*-values of less than 0.05 were considered statistically significant.

## Competing interests

The authors have no actual or potential conflicts of interest to declare.

## Authors’ contributions

Conception and design: MS, XHL, WD. Development of the methodology: XHL, EBZ, FQN, RK. Acquisition of data: KMW, FYJ, TPX. Analysis and interpretation of data: ZJL, RX, JFC. Writing revision of the manuscript: MS, WD, ZXW. Administrative, technical, and material support: XHL, MS. Study supervision: WD, ZXW. All authors read and approved the final manuscript.

## Supplementary Material

Additional file 1: Table S1 Clinicopathological characteristics and BANCR expression of 113 patient samples of NSCLC. Click here for file

Additional file 2: Figure S1 Effects of EZH2, SUZ12 and HDAC3 on BANCR expression. (A) Analysis of BANCR expression levels by qPCR following treatment of SPC-A1 and A549 cells with si-EZH2 and SUZ12.(B) Analysis of HDAC3 mRNA expression levels by qPCR in NSCLC cell lines. HDAC3 expression was upregulated more significantly in SPC-A1, H1650 and H1975 cells compared with 16HBE cells. (C) A549 and SPC-A1 cells were treated with selective HDAC3 inhibitor RGFP966 (dissolved in DMSO) at concentrations of 15μM for 24 hours. Analysis of BANCR expression levels by qPCR following treatment of SPC-A1 and A549 cells with RGFP966. *P < 0.05, N.S. no significant. Click here for file

Additional file 3: Table S2 Sequence of primers and siRNA. Click here for file
