# Supplementary material for: Downregulation of BRAF activated non-coding RNA is associated with poor prognosis for non-small cell lung cancer and promotes metastasis by affecting epithelial-mesenchymal transition
Source: Mol Cancer. 2014 Mar 21;13:68. doi: 10.1186/1476-4598-13-68 (PMC3998010; doi:10.1186/1476-4598-13-68)
Supplement: Additional file 1: Table S1 — Clinicopathological characteristics and BANCR expression of 113 patient samples of NSCLC. [file 1476-4598-13-68-S1.doc]

**Supplementary Table 1** Clinicopathological characteristics and BANCR expression of 113 patient samples of NSCLC

| **Characteristics** | **No. of cases (%)** |
| --- | --- |
| **Age(years)** |  |
| ≤65 | 54(47.8) |
| >65 | 59(52.2) |
| **Gender** |  |
| Male | 68(60.2) |
| Female | 45(39.8) |
| **Histological subtype** |  |
| Squamous cell carcinoma | 68(60.2) |
| Adenocarcinoma | 45(39.8) |
| **TNM Stage** |  |
| Ia + Ib | 34(30.1) |
| IIa + IIb | 38(33.6) |
| IIIa | 41(36.3) |
| **Tumor size** |  |
| ≤5cm | 56(49.6) |
| >5cm | 57(50.4) |
| **Lymph node metastasis** |  |
| Negative | 54(47.8) |
| Positive | 59(52.2) |
| **Smoking History** |  |
| Smokers | 75(66.4) |
| Never Smokers | 38(33.6) |
| **Chemotherapy** |  |
| Yes | 62(54.9) |
| No | 51(45.1) |
| **Expression of BANCR** |  |
| High expression | 53(46.9) |
| Low expression | 60(53.1) |
