# Supplementary figures and images for: Downregulation of BRAF activated non-coding RNA is associated with poor prognosis for non-small cell lung cancer and promotes metastasis by affecting epithelial-mesenchymal transition
Source: Mol Cancer. 2014 Mar 21;13:68. doi: 10.1186/1476-4598-13-68 (PMC3998010; doi:10.1186/1476-4598-13-68)

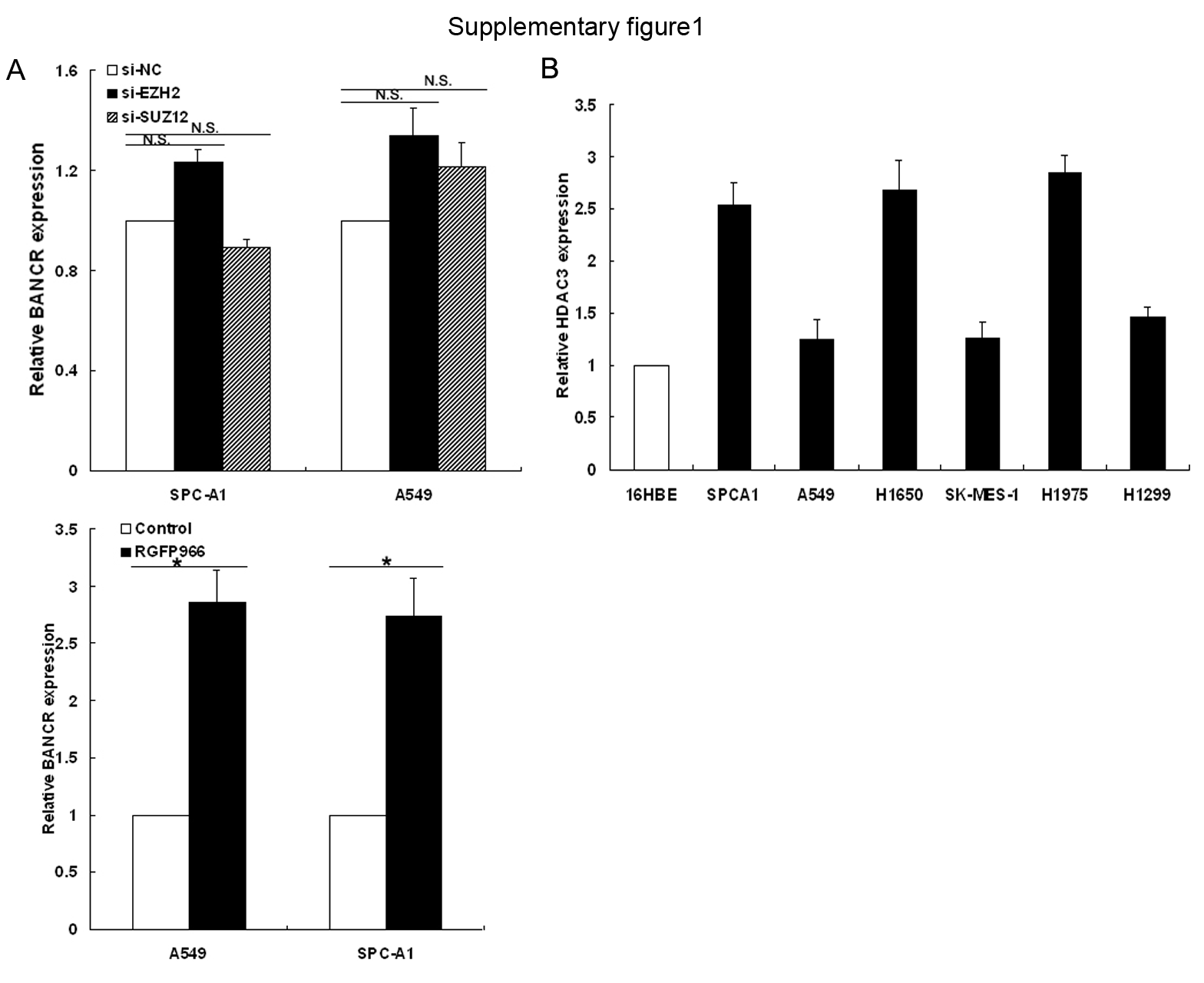

Supplement: Additional file 2: Figure S1 — Effects of EZH2, SUZ12 and HDAC3 on BANCR expression. (A) Analysis of BANCR expression levels by qPCR following treatment of SPC-A1 and A549 cells with si-EZH2 and SUZ12.(B) Analysis of HDAC3 mRNA expression levels by qPCR in NSCLC cell lines. HDAC3 expression was upregulated more significantly in SPC-A1, H1650 and H1975 cells compared with 16HBE cells. (C) A549 and SPC-A1 cells were treated with selective HDAC3 inhibitor RGFP966 (dissolved in DMSO) at concentrations of 15μM for 24 hours. Analysis of BANCR expression levels by qPCR following treatment of SPC-A1 and A549 cells with RGFP966. *P < 0.05, N.S. no significant. [file 1476-4598-13-68-S2.tiff]
